# Mice With Partial Deletion of Y-Heterochromatin Exhibits Stress Vulnerability

**DOI:** 10.3389/fnbeh.2018.00215

**Published:** 2018-09-21

**Authors:** Sandeep Kumar Dey, Avijeet Kamle, Ram Reddy Dereddi, Shiju M. Thomas, Shashi Rekha Thummala, Arvind Kumar, Sumana Chakravarty, Rachel A. Jesudasan

**Affiliations:** ^1^CSIR-Centre for Cellular and Molecular Biology, Hyderabad, India; ^2^CSIR-Indian Institute of Chemical Technology, Hyderabad, India

**Keywords:** Y-deleted mouse, behavior, anxiety, neurogenesis, Y chromosomal homology

## Abstract

The role of Y chromosome in sex determination and male fertility is well established. It is also known that infertile men are prone to psychological disturbances. Earlier studies in the laboratory identified genes expressed in testes that are putatively regulated by Y chromosome in man and mouse. With the availability of a Y-deleted mouse model, that is subfertile, we studied the effect of a partial deletion of Y-chromosomal heterochromatin on mouse behavior when compared to its wild type. The partial Y-deleted mice exhibited anxiety like phenotype under stress when different anxiety (open field test and elevated plus maze, EPM test) and depression related tests (tail suspension and force swim) were performed. The mutant mice also showed reduction in hippocampal neurogenesis and altered expression of neurogenesis markers such as Nestin, *Sox2, Gfap*, *NeuroD1* and *Dcx* using quantitative real time PCR (qPCR) analysis. The genes with altered expression contained short stretches of homology to Y-derived transcripts only in their Untranslated Regions (UTRs). Our study suggests putative regulation of these genes by the Y chromosome in mouse brain altering stress related behavior.

## Introduction

The differences between male and female of any species is well established. They differ with respect to morphology, physiology, behavior and the male/female determining chromosomes (Lovell-Badge, 2005). The male sex hormone androgen can modulate neuronal activity, stem cell self-renewal, differentiation in brain and acts synergistically with genes on Y chromosome to induce aggressive behavior in males. Some Y-chromosomal genes like *Sry* and a few noncoding RNAs are expressed both in testes and in brain (Mayer et al., 2000; Reisert et al., 2002). Such Y-chromosomal genes could affect male behavior. Y-linked genes have been shown to affect rodent brain development and behavior (De Vries et al., 2002; Arnold and Burgoyne, 2004). There are conflicting reports on the effect of Y chromosome on aggressive behavior in mice (Roubertoux et al., 1994; Guillot and Chapouthier, 1998; Sluyter et al., 1999). Roubertoux et al. (1994) observed that pseudoautosomal region of the Y and autosomes had additive effects on the initiation of attack behavior. A number of reproductive behaviors are affected by the male specific region in the Y chromosome (Guillot and Chapouthier, 1998). There are contradictory reports of aggressive behavior in XYY men (Lovell-Badge, 2005). Several studies show that overexpression of Y chromosome leads to the development of aggressive phenotype (Miczek et al., 2001). Individuals with 47, XYY are reported to be at elevated risk for developing anti-social aggressive behavior (Stochholm et al., 2012). A case study on a boy with Attention deficit/hyperactivity disorder (ADHD) showed that a rare deletion of Yq with duplication of Yp made him susceptible to develop ADHD (Liu et al., 2017). De Vries et al. (2002) are of the opinion that much more research is required for strong conclusions and care must be taken to reduce the effects of genetic background. This motivated us to explore whether partial deletion of Y chromosome impacts mouse behavior and susceptibility to neuropsychiatric disorders, using a mutant strain mouse with partial deletion of Y-long arm (XY^RIII^qdel) and its wild type counterpart RIII (XY^RIII^). In general, we noticed that the XY^RIII^qdel mice are hyperactive compared to the wild type, XY^RIII^ animals. Therefore, in the present study Y-deleted (XY^RIII^qdel) male mice were evaluated for their basic mood status, degree of stress responsiveness and also to uncover if it is linked with the hippocampal neurogenesis, which gets affected in rodent models of chronic stress-induced mood disorders (Gould and Tanapat, 1999; Gould and Gross, 2002; Yun et al., 2016).

XY^RIII^qdel mice exhibit severe morphological and motility-related abnormalities of spermatozoa, reduced sperm count and subfertility compared to its wild type, XY^RIII^ (Conway et al., 1994; Touré et al., 2004; Burgoyne and Mitchell, 2007). Yet another strain of Y-deleted mutant strain B10.BR-Y-del shows impaired spermatogenesis and subfertility compared to its wild type, B10.BR/SgSn (Styrna et al., 1991, 2002). Men with microdeletions in the euchromatic long arm of human Y chromosome suffer from varying degrees of infertility (Najmabadi et al., 1996; Vogt et al., 1996). Partial deletion of the Y chromosome also affects reproductive efficiency of females sired by the Y-deleted male (Kotarska and Styrna, 2012; Kotarska et al., 2013, 2015). Over all, most of the reports dealt with and were rather restricted to reproduction related anomalies only, which eventually concluded that deletion of Y chromosome has strong connections with sub-fertility.

Psychological disturbances like anxiety and depression have been reported in infertile men (Sahin et al., 2017; Yang et al., 2017). Sustained grief could lead to major depression as per definition consistent with Diagnostic and Statistical Manual of Mental Disorders, 4th edn, DSM-IV (Ln, 2006). Additionally, if we look through some interesting similarities between the brain and the testes, for instance, both have barriers (the blood-brain barrier and the blood-testes barrier) that restrict the entry of large molecules from the bloodstream, and both organs are very high in cholesterol. In fact, an in-silico analysis indicates that human testis and brain share the highest similarity of gene expression patterns (Guo et al., 2003). However, there is not much information on the regulation by Y-derived transcripts of neural genes involved in behavior, similar to the recently reported putative regulation of testicular autosomal genes by Y chromosomes in mouse, human and *Drosophila* (Vigneault and Zouros, 1986; Jehan et al., 2007; Bhattacharya et al., 2013). In the light of all this information it was legitimate to hypothesize that XY^RIII^qdel mice may be more prone to show stress vulnerability. Using XY^RIII^qdel mice, our study tried to address this aspect too, and we report an interesting finding here. The genes that have altered expression in hippocampal neurogenesis in XY^RIII^qdel mice contain short stretches of homology to Y-derived transcripts in their Untranslated Regions (UTRs). This might have implication for anxiety and related neuropsychiatric disorders.

## Materials and Methods

### Animals

Adult male mice, wild type XY^RIII^ and XY^RIII^qdel mice around 10 weeks old, were used as required in various behavioral and molecular experiments. Animals were obtained originally from Prof. Paul S. Burgoyne, MRC Laboratory, UK and later bred and maintained in Animal House facility of Centre for Cellular and Molecular Biology, Hyderabad, India. The animal room was maintained at 25°C with a 12 h:12 h light-dark cycle (lights off between 18.0 h to 6.0 h). Food and water were available to the animals, *ad libitum*. All animal procedures were carried out in accordance with the approved guidelines of the Institutional Animal Ethics Committee of the Centre for Cellular and Molecular Biology, Hyderabad, India.

### Behavioral Procedure

The mice were subjected to behavior tests that measure anxiety in animals through open field and elevated plus maze (EPM) tests. For all the tests, in-between trials with each mouse, the area was cleaned with 70% alcohol, dried for the next one. A flowchart showing the scheme of experiments has been provided as Supplementary Figure S1.

### Open Field Test

This test was performed according to the previous report in rats (Reddy et al., 2016) with appropriate modifications for mouse. Briefly, each mouse was introduced to the corner of an open field arena (40 × 40 × 40 cm) for this test and was allowed to explore the arena for 5 min. The open-field box floor was virtually divided into 16 equal squares where the central four squares were considered the center zone whereas the twelve peripheral squares were designated as peripheral zone. The amount of time spent in peripheral zone vs. the amount of time spent in the central zone was measured by using video tracking software (Ethovision 3.1 software, Noldus, Netherlands).

### Elevated Plus-Maze Test

This test, to evaluate anxiety status of the animal, was performed with the help of an EPM as reported earlier (LaPlant et al., 2009; Pathak et al., 2017). In detail, EPM consisted of a plus-shaped wooden apparatus elevated at 100 cm above the ground, with two open (33 cm long × 5 cm wide) and two closed arms (with 25 cm tall walls on the sides) and a central region at their intersection. Experimental mice were individually placed in the central region of the EPM and allowed to explore for 5 min, where time spent by mice in each arm was measured using Ethovision 3.1 (Noldus, Netherlands).

### Forced Swim Test

The forced swim test was performed according to previously published protocols (LaPlant et al., 2009; Pathak et al., 2017). Briefly, mice were tested in a 10-L Pyrex glass beaker, filled up to approximately 23 cm with normal water having a temperature of 25 ± 2°C, for 5 min. The entire swimming test session was recorded with a video camera and then was scored manually for the time spent immobile. Total immobility was measured as the time spent without noticeable movement, except for single limb paddling to maintain flotation.

### Tail Suspension Test

The test of tail suspension is also a classic paradigm for assessing depression-like phenotype in mice. It is considered as a “dry” version of usual forced swim test and often used as alternative (Porsolt et al., 2001). Here, mice were individually suspended by means of their tails from a height of around 40 cm from ground for a period of 5 min. Depression was assessed as the measure of total time of immobilization by not trying climbing/escaping from the hanging situation.

### Experimental Stress Paradigm

After conducting all the tests, selected for anxiety and depression in the behavioral battery at basic level, to know how these mice would perform under stress the mice of both groups were subjected to forced swim stress for 10 min for two consecutive days. Twenty-four hours after the last stress episode, we performed again open field test, EPM (for anxiety) and tail suspension test (for depression) to evaluate the degree of response of XY^RIII^qdel mice as compared to wild type (XY^RIII^) mice, under stress.

Before starting first stress episode we have waited for 2–3 weeks to completely diminish the effect of forced swim test as stressor because to check the basal response level of despair behavior we have used the same procedure as one of the behavioral tests. With the same reason keeping in mind that stressor cannot be included as behavioral despair test also, while performing the tests after stress episodes we kept only “tail suspension test” as despair test which is known to be a dry version of behavior despair model (Porsolt et al., 2001; Supplementary Figure S1).

### Brain Tissue Collections

Animals were rapidly decapitated; brains were removed and placed on a sterile platform on ice. Hippocampus (bilateral) was micro-dissected with the help of fine-tipped dissection tools and quickly frozen in liquid nitrogen and stored at −80°C until RNA was extracted.

### BrdU Treatment, Immunostaining and Counting

A separate group of animals (XY^RIII^ and XY^RIII^qdel, 4–5 in each group), were injected with BrdU (50 mg/Kg, intraperitoneally), a thymine analog that gets incorporated in DNA of proliferating cells, twice daily starting 2 days prior to the sacrifice. The mice were intra-cardially perfused with paraformaldehyde and brains were collected and processed for immunostaining as reported earlier (Suri et al., 2013; Chakravarty et al., 2015; Joshi et al., 2017) with minor modifications. In brief, Vectastain ABC kit (Vector Labs) was used and sections were developed with diaminobenzidine (DAB) before mounting in DPX to count BrdU positive cells observed in sub granular zone of hippocampus. Total six sections were used per mouse. Regarding region, approximately following coordinates: anterioposterior = −2 mm from Bregma; lateral = ± 1.6 mm; ventral = 2.5 mm (Ikrar et al., 2013), were used.

### Quantitative mRNA Expression of Neurogenesis Markers Using Real-Time PCR

For molecular study, hippocampal region from XY^RIII^ and XY^RIII^qdel brain tissues were excised out and stored in RNA later (Ambion), until used. The total RNA from each tissue was extracted using Trizol method (Ambion). The purity and quantity of the extracted RNA was checked using Nanodrop (NANODROP 2000, Thermo Scientific) and Qubit (Thermo Scientific). One microgram of isolated RNA was reverse transcribed to cDNA using Verso cDNA synthesis kit (Thermo Scientific). Quantitative Real time PCR (qPCR) was performed using SYBR green master mix (Roche) and analyzed in Roche Light Cycler LC480. The primer sequences corresponding to the neurogenesis markers used are listed in Table 1. The thermal cycling conditions included an initial denaturation for 5 min at 95°C followed by 45 cycles of denaturation at 95°C for 10 s annealing at 58°C for 20 s and elongation at 72°C for 30 s. The amplification of specific product was confirmed by melting curve profile (cooling the sample to 65°C for 1 min and heating slowly with an increase in temperature of 5°C at each step till 95°C, with continuous measurement of fluorescence). The relative fold change in expression was estimated based on Livak method (2^−ΔΔCt^).

**Table 1:** The primer sequences used for real-time PCR studies.

Gene	Primer	Sequence (5′-3′)
*Gapdh*	Forward	tgaagtcgcaggagacaacct
	Reverse	atggccttccgtgttccta
Nestin	Forward	ttgagtggggctgcagctaatgtt
	Reverse	ggggcatctaaatggtcaatcgct
*Sox-2*	Forward	caacgg cag cta cag cat gat
	Reverse	tgc gag tag gacatgctg tag gt
*Gfap*	Forward	agtggccac cag taa cat gcaa
	Reverse	gcgatagtcgttagcttc gtg ctt
*Dcx*	Forward	tctgtttcc cag gcaatgct
	Reverse	aaagggcctgctctaaccagt
*NeuroD1*	Forward	gct act ccaaga ccc agaaactgt
	Reverse	aattgg tag tgggctggg aca aac
*NeuN*	Forward	gcggtc gtg tat cag gat ggattt
	Reverse	atggttccgatgctg tag gttgct

### Statistical Analysis

Mean differences between groups were determined by either a two-tailed unpaired Student’s *t*-test with confidence intervals of 95% or two-way analysis of variance (ANOVA) followed by Bonferroni *post hoc* analysis with confidence intervals of 95%. Data were tested for outliers by means of the Grubbs test before performing ANOVA. This test is based on the difference between the average in a group and the most extreme data point in the group which differs significantly, considering the standard deviation (Grubbs, 1969). For individual variation in each group scatter bar plots were also made and displayed in appropriate places in the figures. Statistical analysis was performed using the software GraphPad Prism 6.0 (San Diego, CA, USA). Results are depicted as means with standard errors (mean ± SEM). Statistical significance was set at *P* < 0.05 “*”, *P* < 0.01 “**” and *P* < 0.001 “***” represents significant difference from the respective controls. “#” represents showing a trend but not significant *P* < 0.09.

## Results

### XY^RIII^qdel Mice Apparently Did Not Show Any Difference in Mood Status Except Slight Hyperactivity

**Figure 1:**
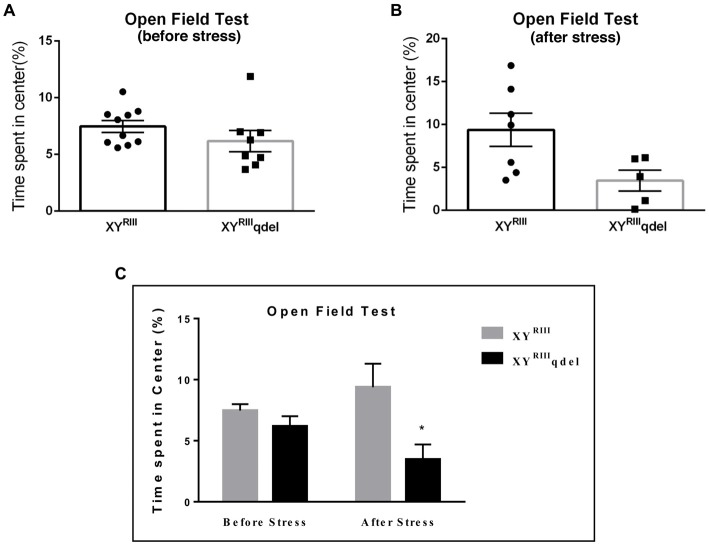
The status of anxiety in XY^RIII^qdel and XY^RIII^ mice was evaluated by open field test before and after the exposure of the stressors. Before the mice have undergone stress paradigm, the status of anxiety did not differ from the wild type as shown by scatter bar plot **(A)**, whereas in the same test they showed significantly more anxiety phenotype by spending remarkably less time in center on stress response **(B)**. Combined bar graph is shown to compare the effect of before and after stress **(C)**. Data are expressed as the mean ± SEM, *P < 0.05, *n* = 8–10 per group in before stress, *n* = 6–8 per group in after stress experiments.

**Figure 2:**
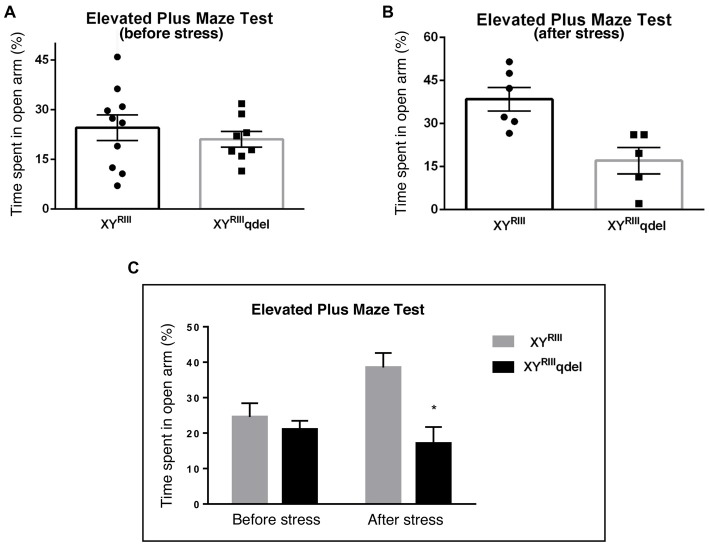
The status of anxiety in XY^RIII^qdel and XY^RIII^ mice was evaluated by elevated plus maze test (EPM) before and after the exposure of the stressors. Before the mice have undergone stress paradigm, the status of anxiety in XY^RIII^qdel did not differ from the wild type as shown by scatter bar plot **(A)** whereas in the same test they showed significantly more anxiety phenotype by spending remarkably less time in open arm on stress response **(B)**. Combined bar graph is shown to compare the effect of before and after stress **(C)**. Data are expressed as the mean ± SEM, *P < 0.05, *n* = 8–10 per group in before stress, *n* = 6–8 per group in after stress experiments.

**Figure 3:**
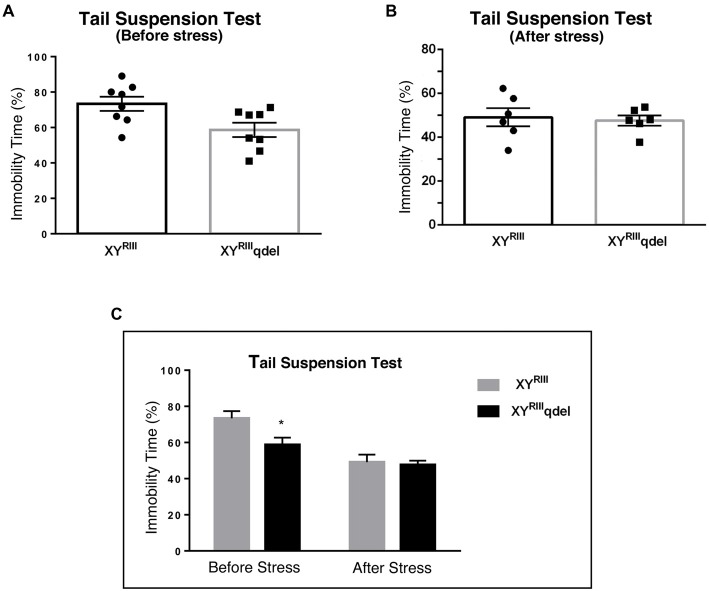
The status of depression in XY^RIII^qdel and XY^RIII^ mice was evaluated by tail suspension test before and after the exposure of the stressors. Before the mice have undergone stress paradigm, XY^RIII^qdel exhibited significant hyperactivity by showing remarkable less immobility time in percentage out of total test period **(A,C)**. Please notice after stress this hyperactivity was leveled as shown by scatter bar plot **(B)** of the same test. Combined bar graph is shown to compare the effect of before and after stress for the same parameter **(C)**. Data are expressed as the mean ± SEM, *P < 0.05, *n* = 8–10 per group in before stress, *n* = 6–8 per group in after stress experiments.

**Figure 4:**
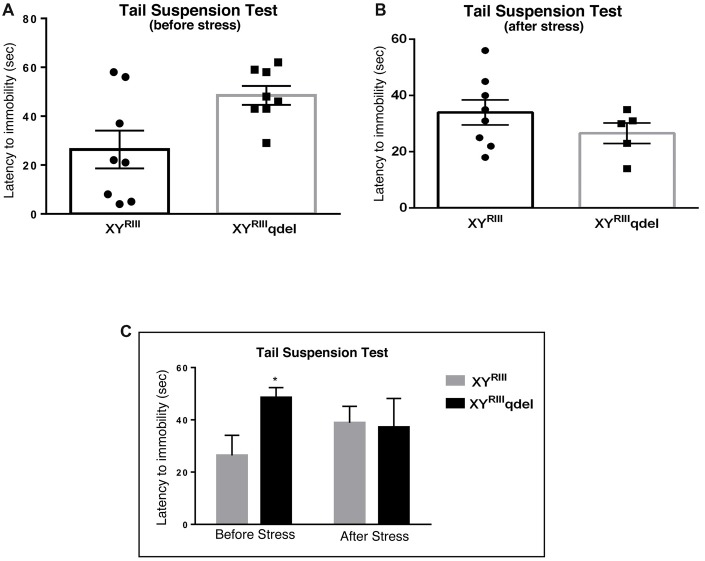
The latency to immobility in XY^RIII^qdel and XY^RIII^ mice was evaluated by tail suspension test before and after the exposure of the stressors. Before the mice have undergone stress paradigm, XY^RIII^qdel exhibited significant hyperactivity by showing remarkably more time in attaining first time immobility **(A,C)**. Please notice after stress this hyperactivity was leveled as shown by scatter bar plot **(B)** of the latency. Combined bar graph is shown to compare the effect of before and after stress in latency **(C)**. Data are expressed as the mean ± SEM, *P < 0.05, *n* = 8–10 per group in before stress, *n* = 6–8 per group in after stress experiments.

To check if there is any basic difference in general mood status of XY^RIII^qdel mice from the XY^RIII^, we performed few specific anxiety (open field test and EPM test) and depression (Tail suspension and forced swim) tests. We did not find any significant change in anxiety level as animals showed no difference in spending time at center in open field test and in open arm of (Figures 1A,C) EPM (Figures 2A,C) test duration. In case of depression specific tests, interestingly we observed in both the despair tests (tail suspension and forced swim tests) XY^RIII^qdel mice showed significantly less immobility percentage as compared to wild type XY^RIII^ (tail suspension test: Figures 3A,C; forced swim test: Supplementary Figures S2A,C) before stress. This might be an indication of hyperactivity of these mice. To confirm this hyperactivity, we precisely checked their latency to get first time immobility and to our surprise we have noticed XY^RIII^qdel mice had significantly higher latency than wild type XY^RIII^ in the despair tests (tail suspension: Figure 4A; however, the forced swim test did not show a clear difference: Supplementary Figures S2B,D).

### XY^RIII^qdel Mice Showed More Vulnerability to Stress by Showing Severe Anxiety (Affective) Phenotype but Not Despair

In order to find out if the XY^RIII^qdel mice differ in their degree of response to stress from their wild type counterpart, the same behavioral tests were repeated after a stress paradigm. Interestingly, we observed XY^RIII^qdel mice developed anxiety phenotype in both the anxiety-specific tests (open field and EPM); they spent significantly less time in open area than the control animals after the stress episodes (open field test: Figures 1B,C and elevated plus test: Figures 2B,C). Beside this, we also found their hyperactivity showing more mobility during despair tests, (Figures 3A,C and Supplementary Figure S2A) also was remarkably reduced under the stress as compared to their wild type control counterparts (tail suspension test: Figures 3B,C). However, forced swim test was not done after stress because we have used the same test protocol as stressor. To take a detail look in their stress-induced amelioration in hyperactivity we carefully observed the latency to immobility for each individual from the videos. We clearly found that there is no visible difference anymore in latency of XY^RIII^qdel mice to attain first time immobility after stress in TST as compared to wild type ones (Figures 4B,C).

### XY^RIII^qdel Mice Failed to Show Any Visible Difference in Locomotion as Such but Exhibited Difference After Stress

**Figure 5:**
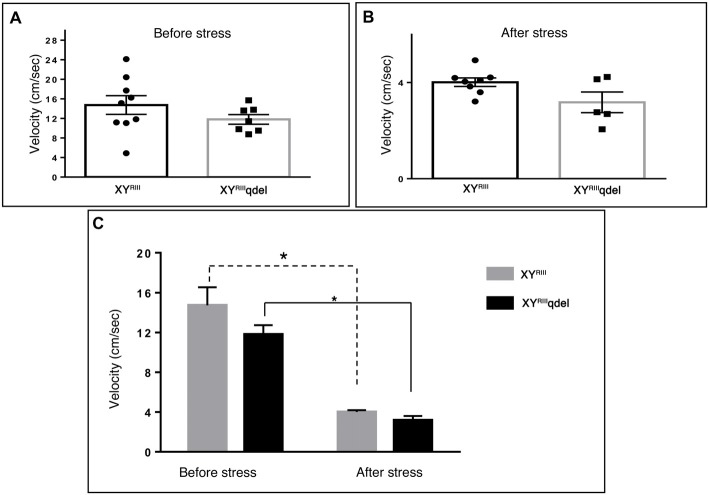
The status of normal locomotion in XY^RIII^qdel and XY^RIII^ mice before and after stress: velocity. Before and after stress the mice did not show any difference in the velocity of their movement as such in between genotypes **(A,B)** whereas while comparing movements before and after stress episodes both the groups have shown significant reduction **(C)**. Data are expressed as the mean ± SEM. Solid line represents the difference between XY^RIII^qdel groups and dotted line represents the wild type before and after stress. *P < 0.05, *n* = 8–10 per group in before stress, *n* = 6–8 per group in after stress experiments.

**Figure 6:**
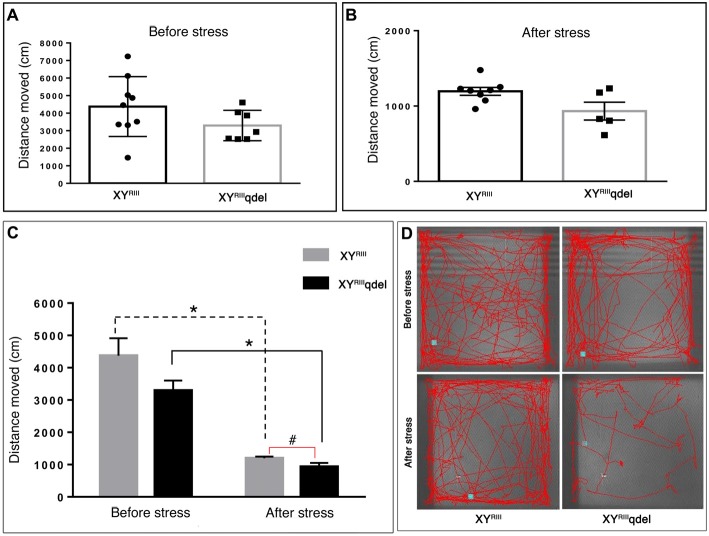
The status of normal locomotion in XY^RIII^qdel and XY^RIII^ mice before and after stress: distance moved. Before and after stress the mice did not show any difference in total distance moved during the test period as such in between genotypes **(A,B)** whereas, after the mice have undergone stress paradigm, XY^RIII^qdel showed a strong tendency of difference from the wild type in distance traveled (*P* < 0.09) **(C)**. Similar to velocity data (Figure 5) on comparison of movements before and after stress episodes both the groups have shown significant reduction in the same parameter. Data are expressed as the mean ± SEM. Solid line represents the difference between XY^RIII^qdel groups and dotted line represents the wild type before and after stress. *P < 0.05, ^#^*P* < 0.09, *n* = 8–10 per group in before stress, *n* = 6–8 per group in after stress experiments. Representative image of animal’s track from each group shown **(D)**.

Once we found an indication that XY^RIII^qdel mice might be having some hyperactivity in the despair tests (Figure 4A and Supplementary Figure S2A), we evaluated their normal locomotor status using open field test area. Unlike TST and FST, we could not notice any significant change in locomotion (neither in velocity: Figures 5A–C nor in distance traveled: Figures 6A–C) of XY^RIII^qdel as compared to wild type ones, however after stress they showed a noticeable reduction in overall distance traveled compared to their counterpart XY^RIII^ mice (Figure 6C). In fact, there was a uniform difference observed in velocity of both the groups of individuals after stress; the XY^RIII^qdel animals were not found to be specifically affected as far as the velocity is concerned (Figures 5B,C). Whereas, after the mice have undergone stress paradigm, XY^RIII^qdel showed a strong tendency of difference from the wild type in distance traveled (*P* < 0.09, #; Figure 6C). The representative tracks of the animal groups before and after stress can be referred for clarity (Figure 6D).

### XY^RIII^qdel Mice Exhibited Poor Hippocampal Neurogenesis

**Figure 7:**
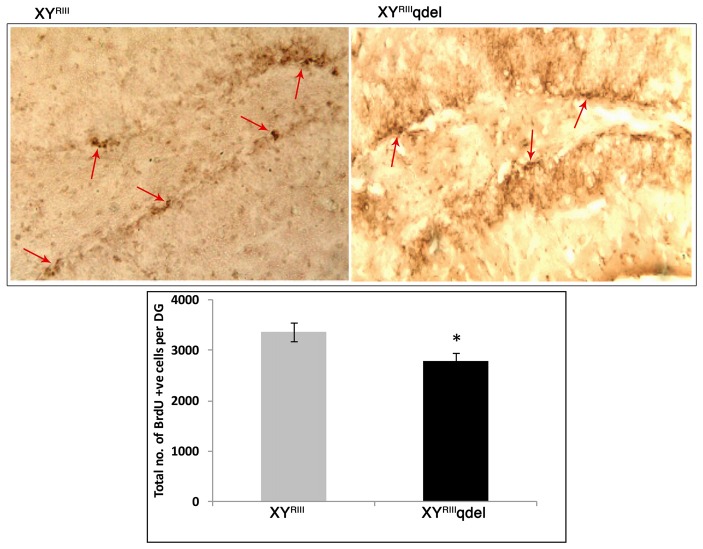
Hippocampal neurogenesis in XY^RIII^qdel and wild type mice. Number of BrdU-positive cells in the sub-granular zone of the dentate gyrus. Upper panel shows representative photomicrographs and lower panel shows the actual counting (*n* = 4–5/group) *P < 0.05.

In order to evaluate the status of basal level of hippocampal neurogenesis, we quantified the number of BrdU positive cells in subgranular layer of dentate gyrus from each mouse. The data suggest that XY^RIII^qdel mice indeed have remarkably reduced turnover of neural stem cells/neural progenitor cells as compared to the XY^RIII^ mice (*P < 0.05; Figure 7). It is pertinent to mention here that before performing the final set of experiments described here, a pilot experiment was performed after FST stress. Unfortunately, after stress (for 2 days) hippocampal neurogenesis of deletion group was reduced to such an extent where it was hard to detect any proliferation (data not shown).

### Proliferative Markers in Hippocampus Also Showed Significant Alteration

**Figure 8:**
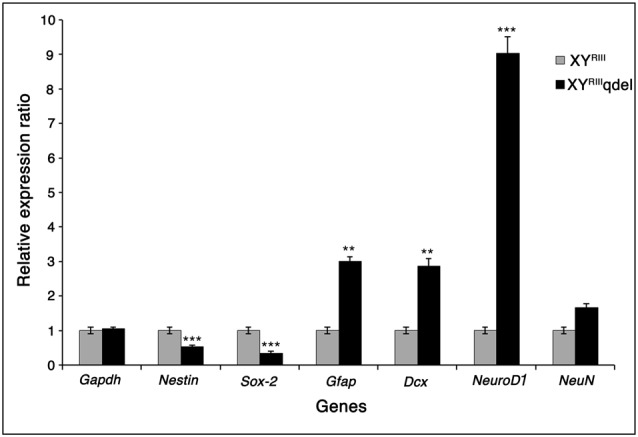
The mRNA expression level of neurogenesis markers. This figure shows the m-RNA expression level of neurogenesis markers in XY^RIII^ and XY^RIII^qdel mice, with reference to *Gapdh*. The proliferation markers Nestin and *Sox-2* (*P* < 0.001, ***) showed significant reduction in expression while the differentiation markers were upregulated in XY^RIII^qdel compared to XY^RIII^ mice. *Gfap* and *Dcx* show upregulation at a significance level of *P* < 0.01, ** and *NeuroD1*, *P* < 0.001, ***). Students *t*-test was used to calculate the statistical significance.

After observing a marked reduction in hippocampal neurogenesis in XY^RIII^qdel mice, we wanted to see if the expression of genes involved in proliferation in the hippocampus also reflect the same without stress. We selected few known proliferation/differentiation gene markers and compared them with a house-keeping gene, *Gapdh*. From the qPCR results, it was observed that the mRNA expression level of proliferation markers nestin and *Sox-2* decreased significantly (*P* < 0.001) in XY^RIII^qdel compared to XY^RIII^, with reference to *Gapdh*. Yet, XY^RIII^qdel mice showed significant increase in the expression of *Gfap* (*P* < 0.01), *Dcx* (*P* < 0.01) and *NeuroD1* (*P* < 0.001), in comparison to XY^RIII^. It was interesting to observe that the *NeuroD1* expression was nine-fold higher in XY^RIII^qdel compared to XY^RIII^. However, no significant change in expression was observed between XY^RIII^ and XY^RIII^qdel for *NeuN*, a marker for mature neurons (Figure 8).

### Y Chromosomal Homology

**Figure 9:**
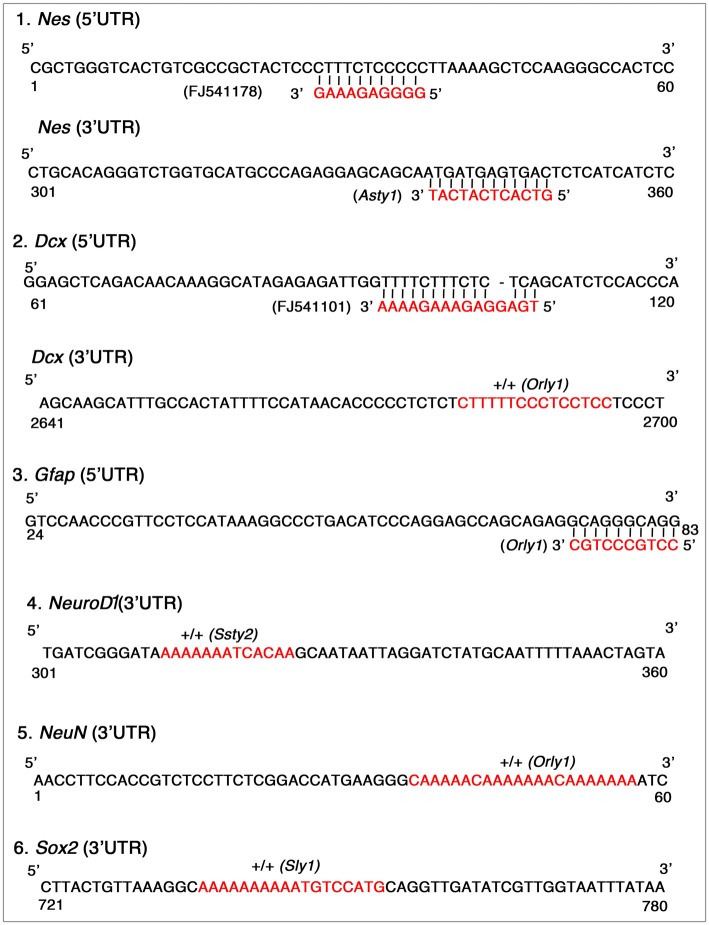
Y-chromosomal homology in untranslated regions (UTRs) of deregulated genes in hippocampus. The short stretches of homology observed between the Y-chromosomal transcripts (red color) and the UTRs of the deregulated genes are represented in the figure. Nestin and *Dcx* had hits in both 5’ and 3’UTRs; *Gfap* had homology only in the 5’ UTR whereas *Sox2*, *NeuroD1* and *NeuN* had unique hits only in their 3’ UTRs.

The six genes studied here in mouse hippocampal neurogenesis, Nestin*, Sox2, Gfap, Dcx, NeuroD1* and *NeuN*, were checked for homology to Y-derived transcripts. The coding and UTR (both 5’ and 3’) sequences of the corresponding genes were obtained from Ensembl. BLASTN of these sequences were performed against the Y-derived transcripts using the parameters of word size 10, E-value 10 with the DUST filter off. All the genes had homology to the Y transcripts specifically in their UTRs (either 5’ or 3’), with no homology in the corresponding coding regions (Figure 9). Nestin and *Dcx* had unique hits in both the UTRs whereas *Gfap* had one unique hit only in its 5’ UTR; *Sox2*, *NeuroD1* and *NeuN* had unique hits only in their 3’ UTRs.

We demonstrate here that mouse Y heterochromatin has defined effects on behavior. A strain of mouse partially deleted in repeats present on the Y chromosome exhibits anxiety phenotype under stress. These mice also have reduced neurogenesis in the hippocampal region. Real time PCR analysis reveals reduced expression of neurogenesis markers and increased expression of differentiation markers. The genes that are deregulated in XY^RIII^qdel hippocampus have small stretches of homology from Y heterochromatin transcripts specifically in their UTRs. Thus, this study elucidates a novel role in behavior for mouse Y-heterochromatin.

## Discussion

We observed behavioral anomalies pertaining to stress in a strain of mouse with partial deletion of Y-heterochromatin. Decrease in hippocampal neurogenesis in the XY^RIII^qdel mice could partially be responsible for the behavioral anomalies observed. This is in line with the established fact in the field that anxiety and related mood disorders are associated with altered hippocampal neurogenesis (Sahay and Hen, 2008; Lee et al., 2013). This decrease in hippocampal neurogenesis is also reflected by significant decrease in the expression of neural stem or progenitor proliferative markers Nestin and *Sox-2* in our study, as shown by others (Lagace et al., 2007b). Decrease in neural stem or progenitor cell proliferation appears to be compensated by increased differentiation of stem or progenitor cells, which is reflected by significant increase in the differentiation markers *Dcx* (early neuronal marker), *Gfap* (glial marker) and *NeuroD1* (transcription factor for differentiation). This could indicate a shift in homeostasis between proliferation and differentiation in hippocampus on deletion of Y-heterochromatin.

The presence of Y-homologous sequences in the UTRs of deregulated genes analyzed in this study could indicate regulation of these genes by Y chromosome. Further this elicits the concept of regulation of hippocampal neurogenesis by Y-heterochromatin. This points to the importance of Y chromosomal transcripts and repeat elements in regulating genes expressed during adult hippocampal neurogenesis. This also substantiates our previous finding that Y chromosome plays a prominent role in regulating autosomal genes (Jehan et al., 2007; Bhattacharya et al., 2013).Our recent molecular data from a study using XY^RIII^ and XY^RIII^qdel mice indicate putative regulation of autosomal genes expressed in testis by Y-derived noncoding RNAs (Reddy et al., 2018).

The behavioral assessment of XY^RIII^qdel mice using a battery of assays showed behavioral changes pertaining to anxiety and hyperactivity. Moreover, they became vulnerable to stress. Since depression and other neuropsychiatric disorders are associated with decreased hippocampal neurogenesis (Boulle et al., 2014; Levone et al., 2015), we anticipate that this might possibly play a major role in making the XY^RIII^qdel mice more susceptible to stress. Our findings are supported by the recent report that the terminal Azoospermia factor (AZFb+c) deletions are associated with abnormal neuropsychiatric condition (Castro et al., 2017). In patients with Yq microdeletions, there is a copy number variation of genes in the Pseudo-autosomal region (PAR; Jorgez et al., 2011). Though the functional role of genes in PAR is less explored, some genes are known to be involved in neuropsychiatric disorders. The Acetylserotonin O-Methyltransferase (*ASMT*) gene located in PAR, which is involved in circadian rhythm abnormalities, is considered as a candidate gene for bipolar depression (Flaquer et al., 2010; Etain et al., 2012). Deletion of *AZFb+c* is found to influence the PAR gene expression, thus altering neuropsychiatric condition (Castro et al., 2017).

There is no sex-specific difference in hippocampal neurogenesis between male and female (Lagace et al., 2007a). But male and female differ in their susceptibility to neuropsychiatric disorders, which suggests direct/indirect involvement of the sex-linked genes in the onset of these brain and behavior disorders (Trent and Davies, 2012). Gene expression in adult human brain has been shown to be different between males and females i.e., the XY and XX genotypes for some genes (Trabzuni et al., 2013). This could be because of the regulation of those genes by Y chromosome in males. Further, we observed a difference in neural gene expression when part of the Y chromosome is deleted. The fact that the genes that are up/downregulated in hippocampus of the XY^RIII^qdel mice exhibit homology to Y-chromosomal transcripts in their UTRs shows a possibility for these genes involved in regulation of hippocampal neurogenesis to be regulated by the Y chromosome. Y-linked genes have been explored for the influence on neural masculinization and their contribution for the sexual differentiation of the brain development and function (Kopsida et al., 2009). The Y-linked genes including the six NRY genes and *Sry* are shown to contribute to neural sexual differentiation (Xu et al., 2002). Our findings would extend the knowledge that apart from the well-established Y-linked genes, there are other possible Y-derived transcripts and repeat elements which could regulate the genes involved in hippocampal neurogenesis and thus affect brain and behavioral function.

The behavioral data in our study shows there are some differences between the wild type and XY^RIII^qdel mice to begin with, i.e., when the animals are not exposed to stress. In unstressed situation, XY^RIII^qdel mice do not exhibit the phenotype of mood related disorders. However, in TST and FST these mutant animals show less immobility, i.e., hyperactivity, compared to their wild type counterparts. Upon stress exposure, the pre-stress level hyperactivity in XY^RIII^qdel mice goes down significantly thus suggesting vulnerability to stress, in addition to increase in anxiety, which the mutants show following stress-exposure.

Regarding the observation that locomotion was significantly reduced in both groups of animals after stress, it is well known that the stress responses differ in acute and chronic stress specially with respect to locomotory behavior in the mouse (Cabib et al., 1988), where they have shown a reduced locomotion on acute stress and increased locomotion after chronic stress (Cabib et al., 1988). Hyper-locomotion also was reported in chronic stress mouse models (Ito et al., 2010). In the present study, we have given 2 days of Forced Swim stressor, which can be considered as “Acute” stress. This could be the reason behind the observation of reduced locomotion in both the groups, where both the groups have undergone the same stress paradigm.

It is well established that chronic long-term stress leads to anxiety and depression. The clinical symptoms of stress, anxiety and depression and its presentation are different in males compared to females (Martin et al., 2013). There is no hormonal ebbs and flows in men, which are the major reasons attributed to behavioral changes in female (Soares and Zitek, 2008). The only hormonal link that is predicted as a possible contributor in neuropsychiatric disorder in males is the association of decreased testosterone level with depression (Kenny et al., 2004). Therefore, there is an urgent need for the identification of male specific regulatory elements, in particular noncoding RNAs in neurodegenerative and neuropsychiatric diseases, for which Y chromosome is a rich source. A deeper understanding of this Y chromosome induced regulation of autosomal genes in brain might also answer why certain neuropsychiatric disorders are gender biased. Our findings might open up new doors for better understanding of neuropsychiatric and neurodegenerative etiology. However, in the light of our behavioral studies, pre-stress hippocampal neuronal gene expression and neurogenesis changes, post-stress gene expression and neurogenesis profiling would have strengthened the behavioral data. Further studies in these lines are warranted for more definite conclusions.

## Author Contributions

SD, AKamle, RD, SMT and SC performed experiments. SRT did bioinformatics analysis. AKumar, SC and RJ conceived the article and wrote the manuscript.

## Conflict of Interest Statement

The authors declare that the research was conducted in the absence of any commercial or financial relationships that could be construed as a potential conflict of interest.
