# Supplementary material for: Mice With Partial Deletion of Y-Heterochromatin Exhibits Stress Vulnerability
Source: Front Behav Neurosci. 2018 Sep 21;12:215. doi: 10.3389/fnbeh.2018.00215 (PMC6160548; doi:10.3389/fnbeh.2018.00215)
Supplement: Supplementary file 1 [file Data_Sheet_1.pdf]

Mice with Partial Deletion of Y-heterochromatin Exhibits Stress Vulnerability

Sandeep Kumar Dey[1,2], Avijeet Kamle[1], Ram Reddy Dereddi[1], Shiju M Thomas[1], Shashi Rekha Thummala[1], Arvind Kumar[1], Sumana Chakravarty[2]*, Rachel A Jessudasan[1]*

[1]CSIR-Centre for Cellular and Molecular Biology, Habsiguda, Uppal Road, Hyderabad 500007, India.[2]CSIR- Indian Institute of Chemical Technology, Taranaka, Uppal Road, Hyderabad 500007, India.

Author for correspondence: Sumana Chakravarty, [2]CSIR- Indian Institute of Chemical Technology, Taranaka, Uppal Road, Hyderabad 500007, India. sumana98@gmail.com; sumanachak@iict.res.in
Rachel A. Jesudasan, CSIR-Centre for Cellular and Molecular Biology (CCMB), Habsiguda, Uppal Road, Hyderabad- 500 007 (T.S.), India.
rachellike@gmail.com; rachel@csirccmb.org
Telephone +91-9440407163
***Co-corresponding authors**

Running Title: Y-deletion and hyperanxiety.

Keywords:
Y-deleted mouse, behaviour, anxiety, neurogenesis, Y chromosomal homology

Supplementary Material for Frontiers.

Supplementary Figure 1

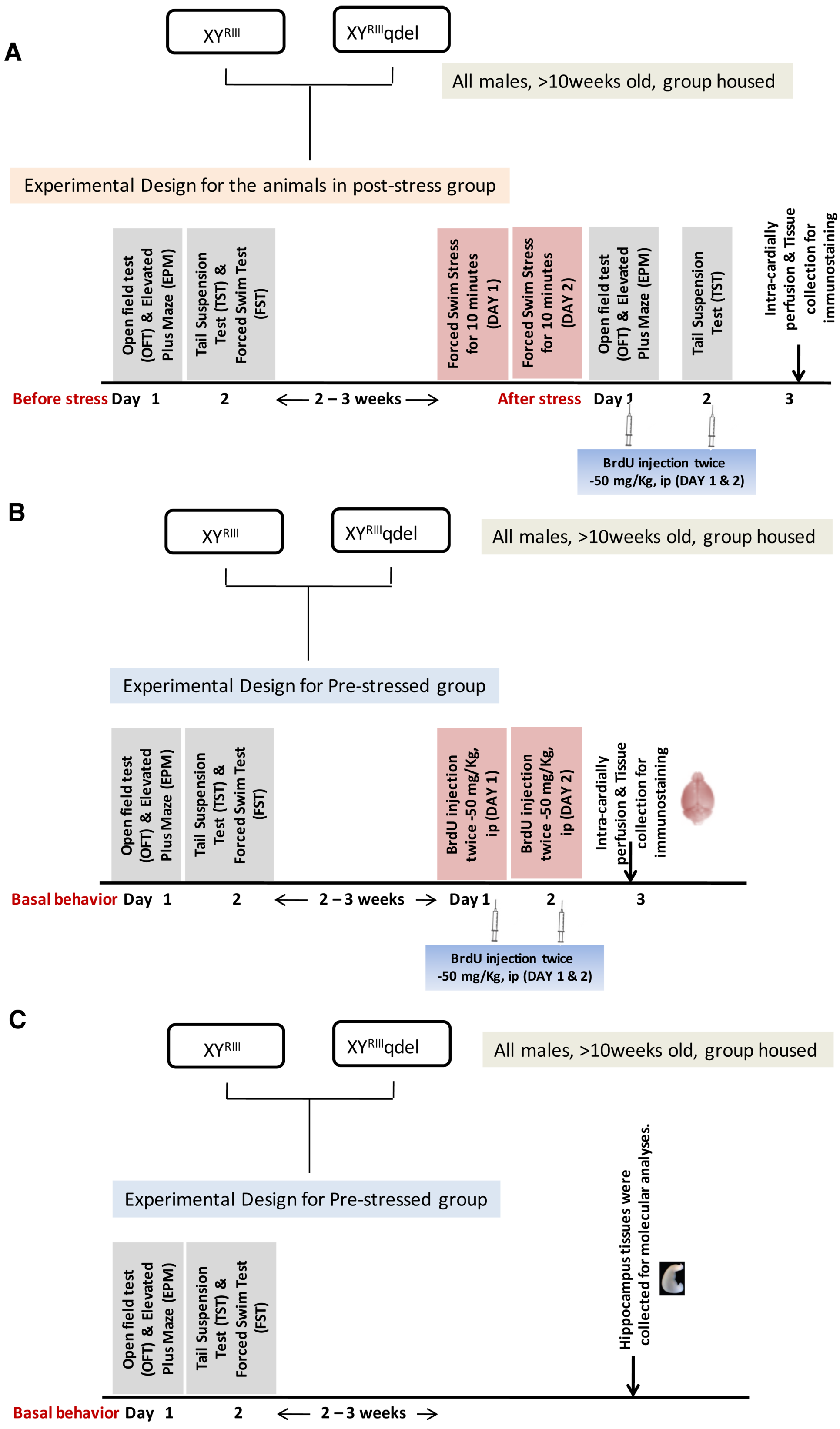

Supplementary Figure 1: Experimental design schematic. **(A)** Stress paradigm used in the post-stress experiment. All animals were group housed. Stressed animals were individually given 2 days of forced swim stress and while doing behavioral tests, the animals were simultaneously injected with BrdU for 2 days, twice daily [50 mg/Kg body weight, intraperitoneally; n=4-8] and on day 3 were collected the hippocampus for molecular analyses.

(B) For pre-stress group after 2-3 weeks of basal behavioral tests the animals were injected with BrdU for 2 days, twice daily [50 mg/Kg body weight, intraperitoneally; n=4-8]. Here animals did not go through the stress paradigm and whole brain was fixed via intra-cardiac perfusion.

(C) same as **(B);** but here animals were not given BrdU injections before the tissues were collected for molecular analyses.

Supplementary Figure 2

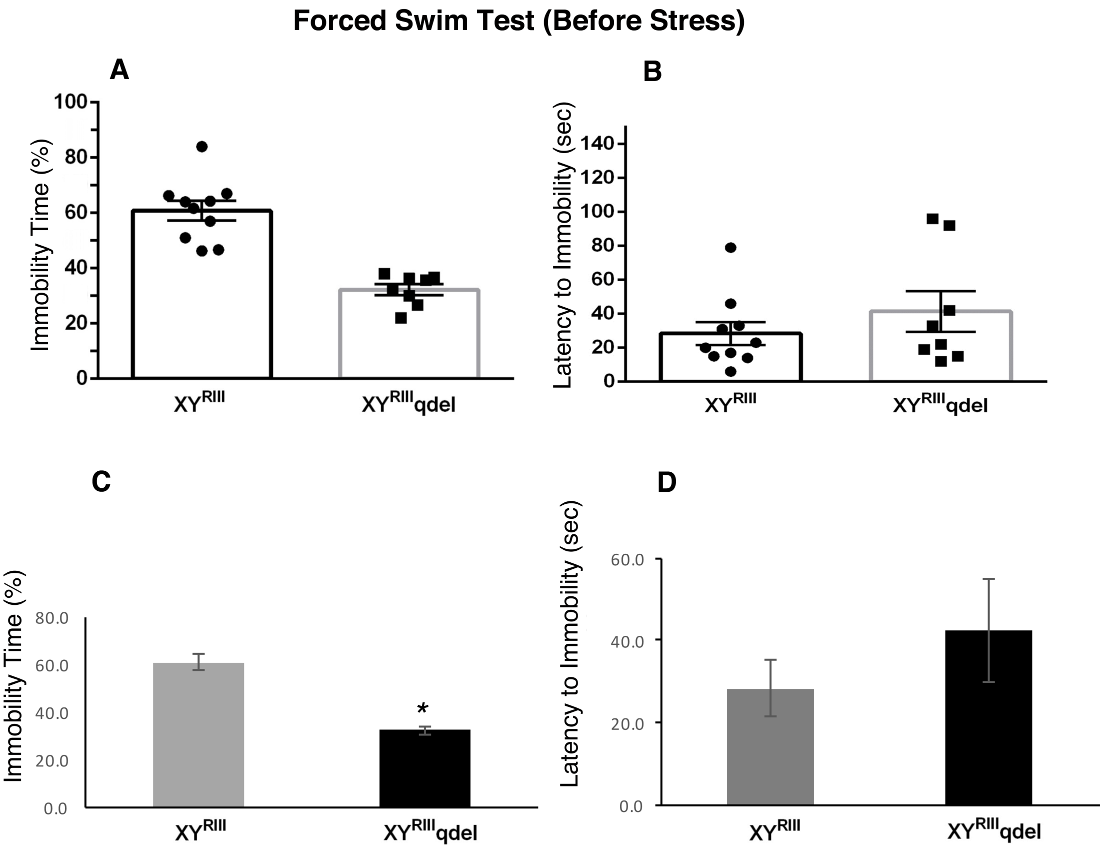

Supplementary Figure 2: The status of depression in XY^{RIII}qdel and XY^{RIII} mice was evaluated by Forced Swim Test before the exposure of the stressors. Before the mice have undergone stress paradigm **XY^{RIII}qdel** exhibited significant hyperactivity by showing remarkable less immobility time in percentage out of total test period (A, C). To show the individual variation the scatter bar plots were made. The latency to attain first time immobility of **XY^{RIII}qdel** was not found to be different from the wild type (B, D). Data are expressed as the mean ± SEM, "*" when $P < 0.05$, n= 8-10 per group in before stress.
